# Household Hints and Recipes

**Published:** 1880-07

**Authors:** 


### Household Hints & Recipes

POWDER FOR SWEATY FEET.

Salicylic acid, 25 parts.
Alum, powd., 50 parts.
Potato flour, 200 parts.
Oil of Bergamot, 10 parts.
Alcohol, 50 parts.
Talc, powd., 700 parts.

---

PROTECTION OF IRON CASTINGS.—Rub 1 part of graphite to a powder, add 4 parts of sulphate of lead, 1 part of sulphate of zinc, and 19 parts of linseed oil varnish ; mix well and boil. This forms a varnish which no weather will wash off.

---

MAKING BOOTS WATERPROOF.—Linseed oil, 1 part ; mutton tallow, ½ lb. ; melt and mix thoroughly together, and apply to the warm, dry leather with a brush. A small quantity of ivory black is sometimes added to this mixture. —*Scien. Amer.*

---

LIQUID GLUE.—Very strong glue may be made by dissolving 4 oz. of glue in 16 oz. of strong acetic acid by the aid of heat. It is semi-solid at ordinary temperature, but needs only to be warmed, by placing the vessel containing it into hot water, to be ready for use.

---

TOBACCO SMOKE FOR GAPES IN CHICKENS.—A correspondent of the *Rural New Yorker* recommends tobacco smoke to cure gapes in chickens. A chicken which was nearly dead with the disease, on being treated by inhalation of tobacco smoke until it was stupified, was cured in one day.

---

TO CLEAN CELLULOID COLLARS.—"When soap, pumice stone, and tripoli fail to remove the yellow tinge often produced by perspiration, I find that a good way is to take a small piece of sponge or cloth, to saturate it with sulphuric ether, and to rub it on the parts that are colored or tarnished.

---

GUM EUPHORBIUM AS A COATING FOR SUBMERGED IRON. Attention is called by *Iron* to the value of the gum euphorbium for coating iron exposed to the action of sea-water. It not only, by its poisonous properties, prevents the adhesion of marine animals, but also keeps the iron from rusting.—*Pharm. Jour. and Trans.*

REMEDY FOR CORNS.—Mr. Gezow, a Russian apothecary, recommends the following as a "sure" remedy for corns, stating that it proves effective in a short time, and without causing any pain :

Salicylic acid, 30 parts.
Extract of cannabis indica, 5 parts.
Collodion, 240 parts.

To be applied by means of a camel's-hair pencil.—*Pham. Zeit. f. Rassel.*,

---

POSTAGE STAMP MUCILAGE.—The mixture used on the U. S. postage stamps is stated to be as follows : 2 parts of dextrin are dissolved in one part of acetic acid and 5 parts of water on a water-bath, and after solution, 1 part of alcohol is added.

In Germany, only gum arabic is used for postage stamps. According to the *Droguisten Zeitung*, the amount daily consumed by the Imperial Post Office Department is said to be 100 pounds.

---

A GOOD BLACKING.—Probably the best ordinary blacking which can be produced is made by the following formula, which has been long in use :

Mix intimately 1 pound of molasses, 1 pound of best bone-black in *very* fine powder, and ¼ pound olive oil ; then add ¼ pound sulphuric acid, previously diluted with ¾ pounds of water. The whole is allowed to stand for 3 hours or longer, and afterwards as much water is added as is necessary to give it the proper consistency.

---

GOOD PASTE, VERY CHEAP.—

Carpenter's glue, in small pieces, 1½ dr'ms.
Pearl starch, in powder, 6 drachms.
Water, 8 ounces.

Dissolve the glue by heat in half the water, add to it the starch, previously made into a thin paste, with the remainder of the water, and boil the whole until clear, stirring constantly. Add twenty drops of the caustic solution of carbolic acid, to preserve, and stir the paste occasionally while cooling to make it smooth. The product never becomes watery or sour, but has always a good smooth body until it dries up. The substitution of a little glycerine for some of the water, and the precaution of keeping the paste in a closed vessel, will greatly retard its drying, and it will keep good for a long time. For general use, however, the glycerine will not at all be necessary.

TURNER'S CEMENT.—Melt 1 lb. of resin in a pan over the fire, and, when melted, add ¼ of a lb. of pitch. While these are boiling, add brick dust until, by dropping a little on a cold stone, you think it hard enough. In winter it may be necessary to add a little tallow. By means of this cement a piece of wood may be fastened to the chuck, which will hold when cool; and, when the work is finished, it may be removed by a smart stroke with the tool. Any traces of the cement may be removed from the work by means of benzine.

KEROSENE OIL LAMPS.—The cement commonly used for fastening the tops on kerosene lamps is plaster of Paris, which is porous and quickly penetrated by the kerosene. Another cement which has not this defect is made with three parts of resin, one of caustic soda and five of water. This composition is mixed with half its weight of plaster of Paris. It sets firmly in about three-quarters of an hour. It is said to be of great adhesive power, not permeable to kerosene, a low conductor of heat, and but superficially attacked by hot water.

IRON CEMENT FOR CLOSING THE JOINTS OF IRON PIPES.—Take of coarsely powdered iron borings, 5 pounds ; powdered sal-ammoniac, 2 oz. ; sulphur, 1 oz. ; and water sufficient to moisten it. This composition hardens rapidly; but if time can be allowed, it sets more firmly without the sulphur. It must be used as soon as mixed and rammed tightly into the joint.

2. Take sal-ammoniac, 2 oz. ; sublimed sulphur, 1 oz. ; cast-iron fillings or fine turnings, 1 lb. Mix in a mortar and keep the powder dry. When it is to be used, mix it with twenty times its weight of clean iron turnings, or filings, and grind the whole in mortar ; then wet it with water until it becomes of convenient consistence, when it is to be applied to the joint. After a time it becomes as hard and strong as any part of the metal.

SUGAR FROM SORGHAM.—Dr. Collyer, chemist of the Agricultural Department, is confident that one-tenth of the corn acreage of Illinois would suffice to raise all the sugar used in the United States, if devoted to sorgham of the variety best suited to the latitudes allowing practical results to reach not only 50 per cent. of those obtained in his most favorable experiments. The cost of the raw sugar, he thinks, should not exceed three cents a pound. The early amber cane is the species best suited to Illinois. Commissioner Le Duc, on returning from a tour of inspection in the West, reported that the most promising results have already been obtained. He visited one manufactory in Illinois, where 43,000 lbs. of sorgham sugar were made last season, equal in every respect to the best product of the sugar cane ; and this enterprise has been carried on under exceptional difficulties. He visited or received reports from many other localities to which he had sent sorgham seeds, all speaking in the most favorable terms of the prospects.—*Scien. Amer.*

HOW TO MEDICATE A PIG.—At a recent meeting of an English farmer's club, Prof. McBride spoke of the difficulty of administering medicine to a pig. He said: To dose a pig, which you are sure to choke if you attempt to make him drink while he is squealing, halter him as you would for evecution, and tie the rope end to a stake. He will pull back until the rope is tightly strained. When he has ceased his uproar and begins to reflect, approach him and between the back part of his jaws insert an old shoe, from which you have cut the toe leather. This he will at once begin to suck and chew. Through it pour medicine, and he will swallow any quantity you please.—*Scien. Amer.*

PURIFYING HONEY.—Pour the honey into a perfectly clean cylindrical vessel, with straight sides, rather narrow, and having a small lip at the open margin, and heat the vessel on a water-bath. When the contents is hot, pour enough honey into the vessel to fill it, within about 1 inch of the edge, and allow it to remain at rest in the water-bath, at a moderate heat, for about one hour. During this time most of the impurities will rise to the top, while some other may sink to the bottom. Now remove the vessel very carefully from the water-bath, and pour on top of the hot honey, very gently, a sufficient amount of cold water to fill the vessel completely. This will cause all the impurities floating on the honey at once to rise to the top of the cold water, where they will often solidify to a tough skin or cake, which may be taken off without difficulty. Then pour off the water through the lip, remove the last remnants, if necessary, by means of blotting paper, and filter the honey through a piece of well-washed, wetted, and dense white flannel. The resulting product—if the honey was pure—will be very nice and brilliant.

